# Succinate Coenzyme A Ligase Beta-Like Protein from *Trichinella spiralis* Suppresses the Immune Functions of Rat PBMCs In Vitro and Inhibits the Secretions of Interleukin-17 In Vivo

**DOI:** 10.3390/vaccines7040167

**Published:** 2019-11-02

**Authors:** Xiaoke Sun, Yin Li, Muhammad Ali-ul-Husnain Naqvi, Sana Zahra Naqvi, Wen Chu, Lixin Xu, Xiaokai Song, Xiangrui Li, Ruofeng Yan

**Affiliations:** MOE Joint International Research Laboratory of Animal Health and Food Safety, College of Veterinary Medicine, Nanjing Agricultural University, Nanjing 210095, China; 2016107072@njau.edu.cn (X.S.); muziyin08@163.com (Y.L.); 2017207047@njau.edu.cn (M.A.-u.-H.N.); 2018207077@njau.edu.cn (S.Z.N.); 2017107076@njau.edu.cn (W.C.); xulixin@njau.edu.cn (L.X.); songxiaokai@njau.edu.cn (X.S.); lixiangrui@njau.edu.cn (X.L.)

**Keywords:** SUCLA-β, *Trichinella spiralis:* rat PBMCs, immunomodulatory, IL17, cytokines

## Abstract

Succinate Coenzyme A ligase beta-like protein (SUCLA-β) is a subunit of Succinyl-coenzyme A synthetase, which is involved in substrate synergism, unusual kinetic reaction in which the presence of SUCLA-β for one partial reaction stimulates another partial reaction. *Trichinella spiralis* is a parasitic nematode, which may hinder the development of autoimmune diseases. Immunomodulatory effects of SUCLA-β from *Trichinella spiralis* in the parasite-host interaction are unidentified. In this study the gene encoding *T. spiralis* SUCLA-β was cloned and expressed. Binding activities of recombinant *T. spiralis* SUCLA-β (rTs-SUCLA-β) to rat peripheral blood mononuclear cells (PBMCs) were checked by immunofluorescence assay (IFA) and the immuno-regulatory effects of rTs-SUCLA-β on cell migration, cell proliferation, nitric oxide (NO) production and apoptosis were observed by co-incubation of rTs-SUCLA-β with rat PBMCs in vitro, while cytokine secretions in rTs-SUCLA-β treated rats were evaluated in vivo. Furthermore, phagocytosis of monocytes was detected by flow cytometry and effects of rTs-SUCLA-β-induced protective immunity on *T. spiralis* adult worms and muscle larva were evaluated in rats. The IFA results revealed that rTs-SUCLA-β could bind to rat PBMCs. Treatment of PBMCs with rTs-SUCLA-β significantly decreased the monocyte phagocytosis, cell migration and cell proliferation, while NO production and apoptosis of PBMCs were unaffected. Results of the in vivo study showed that the IL-17 secretion decreased significantly after rTs-SUCLA-β administration in rats, while no significant effects were observed on the secretions of IFN-γ, IL-9, TGF-β and IL-4. Moreover, significant reduction of *T. spiralis* muscle larvae burden and significant increase in anti-rTs-SUCLA-β immunoglobulin level of IgG, IgG1 and IgG2a was observed in rTs-SUCLA-β-administered rats. The results indicated that rTs-SUCLA-β may be a potential target for controlling *T. spiralis* infection by suppressing the immune functions of the rat PBMCs and by reducing the parasite burden. Additionally it may also contribute to the treatment of autoimmune diseases and graft rejection by suppressing IL-17 immune response in the host.

## 1. Introduction

The immune system is a defense mechanism in the body that involves peripheral blood mononuclear cells (PBMCs). The function of the immune system is to abolish stimuli including pathogenic and nonpathogenic microorganisms and parasites that have assaulted the body and mutation produced cancer cells [1]. In chronic inflammatory reactions excessive elimination of targets may cause autoimmune diseases. Parasitic nematodes have developed complex mechanisms to contribute in host immunomodulation [2]. Although parasitic infections are considered as hazard for human health, but different animal model systems showed reduced autoimmunity in association with parasite-induced infection [3]. 

*Trichinella spiralis* (*T. spiralis*) is a zoonotic nematode that causes chronic infection in a wide range of mammalian hosts [4]. It can hinder development of autoimmune diseases and other inflammatory disorders by creating an anti-inflammatory environment [5]. It is also an important parasite for the following reasons. First, it is causative agent of human disease, trichinellosis. Second, it is also prevalent in many different domestic animals and last, it provides high infectivity for laboratory animals to perform basic immunological, pathological and biological in vivo models studies [6]. *T. spiralis* is unique among other parasites as it spent all development stages of its life cycle (infective muscle larvae, adult and new born larvae) within one host. Infection is acquired by intake of infected, undercooked or raw meat; larvae are released by the influence of gastric juices in the stomach and develop into adult within the enterocytes of small intestine after molting. Newborn larvae are released after mating and spread throughout the organs and tissues by the circulatory system. The only larvae those enter the striated muscles develop into mature muscle larvae. *T. spiralis* has ability to build their home within the body of host by transforming infected muscle cells into new type of cells, the so-called nurse cell [7,8]. Intracellular localization of these parasites takes place at skeletal muscle cells and enterocytes, which are known as habitat of this parasite [9]. Control and prevention of trichinellosis depend upon success of preventive measures. However, because of economic hurdles in these preventive measures, a vaccine for veterinary use is best alternative to prevent humans by adding in animal feed.

Different nematodal immunomodulatory molecules have been proposed as a vaccine candidate that influences the cytokines function, which may be involved in the protection of the host from parasitic infection [10]. T helper (Th) cells play important role in the eradication of foreign microorganisms and in self-tolerance. Th17 cells produce interleukin (IL)-17 cytokine that is an important cytokine for protective immunity against extracellular as well as intracellular pathogens and plays an important role in the pathogenesis of various autoimmune inflammatory diseases [11]. Th9 cells also promoted the development of autoimmune and allergic diseases by producing IL-9 [12]. IL-9 is promoted by IL-4 and TGF-β (transforming growth factor-beta) while production of both IL-9 and IL-17 is associated with the blocking of IFN-γ and IL-4 cytokines [13]. Previously antigen-induced IL-17 response in PBMCs of healthy controls was investigated [14]. Recently, a study has been conducted which reported the production of Th17 cells in the absence of TGF-β signaling in vivo [15]. Immunologically, *T. spiralis* muscle larvae (ML) excretory and secretory products (ESPs) provide better opportunities to build long lasting communication between host and parasite [16]. The study of these molecules is crucial to understand the parasitism mechanism, novel therapies development and treatments for inflammatory diseases [9]. Potential relationships between parasite ESPs and host cytokines have been reported in recent studies [2,17,18].

Succinate Coenzyme A ligase beta-like protein (SUCLA-β) is one of the important *T. spiralis* ML ESPs that belongs to the Succinyl-coenzyme A synthetase gene and plays an important role in the citric acid cycle [19]. It shows an unusual kinetic reaction termed as substrate synergism, where by the presence of SUCLA-β for one partial reaction stimulates another partial reaction [20]. The binding of SUCLA-β complex with nucleoside diphosphate kinase may be important in the pathogenesis of mitochondrial DNA depletion. The *SUCLA-β* gene is expressed primarily in the skeletal muscle and brain and primarily defects associated with this gene are myopathy and encephalopathy [21]. Due to these characteristics SUCLA-β may have immunomodulatory potential and in our previous comparative proteomics study (data not published), expression level of Ts-SUCLA-β in the ML stage was up-regulated by more than 100 times when compared with newborn larvae (NBL) indicating that Ts-SUCLA-β might be a larval invasion-related protein. However, molecular cloning was not conducted and functional descriptions of *T. spiralis* SUCLA-β were not provided in that study.

Present study was designed to evaluate the different immunomodulatory effects of SUCLA-β from *T. spiralis* on PBMCs in vitro and cytokines secretions in rat. Firstly, the open reading frame (ORF) of the *SUCLA-β* gene was cloned then expressed in an expression vector system and immuno-reactivity was confirmed by immunoblotting. Moreover, immune protection generated by the rTs-SUCLA-β immunization was also evaluated in rats.

## 2. Materials and Methods

### 2.1. Animals and Parasites

ICR (Institute of Cancer Research) mice (body weight 18–20 g) were purchased from the Experimental Animal Center of Jiangsu, P. R. China (Qualified Certificate: SCXK 2008–0004) and kept under controlled conditions at the Animal house of Nanjing Agricultural University. The isolate of *T. spiralis* (ISS534) used in this study was taken from a pig in Nanyang city, Henan Province, China and maintained by serial passage in ICR mice every 6–8 months. Muscle larvae were recovered from *T. spiralis* infected mice by euthanizing at 35^th^ post infection day, the carcasses was digested artificially using digestion solution (0.33% pepsin and 1% HCl) at 43 °C and muscle larvae were collected after 2 h [22,23]. Furthermore, SD rats having 150–160 g body weight were also purchased from center mentioned above to use in further experiments. 

### 2.2. Serum Samples

To collect the serum samples for detection of antibody responses, ICR mice were divided into two groups, experimental group (*n* = 3) and control group (*n* = 3). Mice of experimental group were orally infected with 300 *T. spiralis* ML and the control group was kept uninfected. At the 20^th^ day of infection mice of the experimental group were anesthetized and serum samples were collected to detect the specific antibodies [22]. Furthermore, serum samples from uninfected mice were also collected (control). After collecting blood samples, mice were euthanized by head dislocation.

### 2.3. Cloning and Sequence Analysis of SUCLA-β Gene

Total RNA was extracted from ML using Trizol reagent (Invitrogen, Shanghai, China) [24]. The complementary (c)DNA was synthesized using cDNA Kit (Takara Biotechnology, Dalian, China) as per manufacturer’s instructions and stored at –20 °C until use. The complete ORF (open reading frame) of SUCLA-β was amplified by RT-PCR (reverse transcription-polymerase chain reaction) using specific primers designed from CDS (conserved domain sequences) of *T. spiralis* (Gene bank: KRY28749.1). The sequence of specific primers carrying *BamH* I and *Xho* I digestion site is as following: Sense Primer: (5′-CGCGGATCCATGGCAGCTGCATGGATACCACGAT-3′) and antisense primer: (5′-CCGCTCGAGGATAGGTAGTTCGAAACTGACATCAAGCG-3′). The total 25 μL PCR product was made up of 12.5μL 2 × Taq Master Mix (Takara Biotechnology), 2 μL of cDNA, 8.5 μL ddH_2_O and 1 μL of each primer. PCR amplification was performed as follows: Initial denaturing (one cycle) 94 °C for 5 min, followed by 35 cycles of 94 °C for 30 s, 55 °C for 30 s and 72 °C for 1–2 min, finally extension at 72 °C for 10 min. E.Z.N.A. Gel Extraction Kit (Omega Bio-tech, Norcross, GA, USA) was used according to instructions of manufacturer to purify the PCR products and followed by ligation into cloning vector, pMD19-T (Takara, Dalian, China). Transformation of recombinant plasmid (pMD19-T/SUCLA-β) into *Escherichia coli* DH_5α_ strain (Invitrogen Biotechnology, Shanghai, China) was performed and cultured in ampicillin containing Luria Bertini (LB) medium. The recombinant plasmid, pMD19-T/SUCLA-β was identified by restriction enzyme digestion with *BamH* I and *Xho* I. SUCLA-β gene was sub-cloned into pET-32a, followed by confirmation of successful insertion in the proper reading frame by sequencing (Invitrogen Biotechnology) and blast online. 

### 2.4. Expression and Purification of Recombinant SUCLA-β

The recombinant plasmid of Ts-*SUCLA-β* gene (pET-32a recombinant with Ts-SUCLA-β) was transformed into *E. coli* BL21 and cultured in ampicillin (100 μg/mL) containing LB at 37 °C. Protein was expressed by adding 1 mM isopropyl-β-d-thiogalactopyranoside (IPTG; Sigma-Aldrich, Shanghai, China) until the optical density (OD) 600 of the culture reached 0.6 [24] The cells were collected by centrifugation and lysed using lysozyme (10 μg/mL; Sigma-Aldrich) and sonicated. The cell sonicated product was analyzed by 12% (w/v) sodium dodecyl sulfatepolyacrylamide gel electrophoresis (SDS-PAGE). The recombinant Ts-SUCLA-β (rTs-SUCLA-β) protein was purified by Ni2+-nitrilotriacetic acid (Ni-NTA) column according to the manufacturer’s instructions. After that, the His-tagged protein was washed by elution buffer (300 mM NaCl, 40 mM NaH2PO4, pH 8.0) containing 400 mM of imidazole. The purity of purified rTs-SUCLA-β was analyzed by 12% SDS-PAGE followed by Coomassie blue staining. The purified rTs-SUCLA-β was refolded by a renaturation buffer (20 mmol/L Tris-Cl, 500 mmol/L NaCl, 1 mmol/L GSH, 0.1 mmol/L GSSG, pH 8.0) containing different concentrations of urea (8, 6, 4, 2 and 0 M) [25]. The concentration of refolded rTs-SUCLA-β protein was determined by Bradford procedure [26]. The rTs-SUCLA-β protein was detoxified using ToxinEraserTM Endotoxin Removal Kit (GeneScript, Piscataway, NJ, USA)

### 2.5. Generation of Polyclonal Antibodies

To generate polyclonal antibodies against rTs-SUCLA-β, SD rats (*n* = 6) were divided into two groups, the experimental group (*n* = 3) and control group (*n* = 3). Recombinant Ts-SUCLA-β protein (0.3 mg) was mixed equally with Freund’s complete adjuvant and subcutaneously injected to experimental group. After two weeks, three more doses of protein mixed equally with Freund’s incomplete adjuvant were injected with 1 week interval. Sera samples from control group and experimental group were collected after 10 days of last immunization and stored at –80 °C until further use. 

### 2.6. Immunoblotting Analysis

Immunoblotting analysis was performed to confirm the specific reactivity in sera of experimentally infected mice and to detect *T. spiralis*-specific antibody responses in sera of rTs-SUCLA-β treated rats as described previously [27]. Briefly, the purified rTs-SUCLA was first deposited in SDS-PAGE wells individually, and then transferred onto PVDF (polyvinyl difluoride membrane, Millipore, Billerica, USA) with the help of semi dry system (Hoefer, Holliston, USA) and transfer solution (Tris 48 mM, glycine 39 mM, SDS 0.0375%, methanol 20%). The membrane was blocked with 5% skimmed milk diluted in TBS-T (Tris-Buffered Saline containing 0.05% Tween 20) for 2 h at 37 °C. The PVDF membrane was cut, washed (three times) and incubated with anti-*T. spiralis* 1:300 diluted positive (experimental infected mice/immunized rats and) and negative (uninfected mice/ un-immunized rats) serum for 2 h at 37 °C. Following three washes with TBS-T the strips were incubated with 1:2000 diluted secondary antibodies, horseradish peroxidase (HRP)-conjugated goat anti-mice IgG (Sigma, St. Louis, MO, USA). Subsequently, the strips were washed five times and immunoreactions were visualized using freshly prepared diaminobenzidine (DAB, Sigma, St. Louis, MO, USA)

### 2.7. Separation of Rat PBMCs 

Blood of SD rat was collected into anticoagulant (7.2 mg dipotassium EDTA) containing tube and PBMCs were separated on Ficoll-Histopaque (GE Healthcare, USA) by standard gradient centrifugation method described previously [28]. After centrifugation, cells were washed with PBS (phosphate-buffered saline) and suspended in RPMI (Roswell Park Memorial Institute) 1460 medium (Sigma–Aldrich, St Louis, MO, USA) complemented with penicillin (100 U/mL), 10% heat inactivated fetal bovine serum (ThermoFisher, Waltham, USA) and streptomycin (100 mg/mL; GIBCO, UK). Trypan blue exclusion test was performed to evaluate the cell viability as described previously [29]. The cells were adjusted to the density of 1 × 10^6^ cells/mL as reported previously [30]. 

### 2.8. PBMCs Binding Assay

Immunofluorescence assay was performed to evaluate the binding ability of rTs-SUCLA-β to SD rat PBMCs as described previously [24]. Briefly, freshly rat PBMCs were inoculated with 10 μg/mL rTs-SUCLA-β and 10 μg/mL purified pET-32a protein separately in a 24-well plate (1 mL/well). Plate was incubated at 37 °C in a humidified atmosphere with 5% CO_2_ for 1 h. Washed cells were permitted to settle on poly-L-lysine coated glass slides for 20 minutes and fixed with 4% phosphate-buffered paraformaldehyde at room temperature for 30 minutes. The slides were blocked with PBS containing 5% BSA at 37 °C for 1 h. Subsequently, slides were incubated with 1:100 dilution primary antibodies, rat anti-*T. spiralis* sera and normal sera (control slide) for two hours. After three washes slides were incubated in dark with secondary antibody, goat anti-rat IgG coupled with Cy3 (Beyotime Institute of Biotechnology, Shanghai, China; 1:1000 dilutions) for 30 minutes. DAPI (Sigma, St. Louis, MO, USA) was subsequently added and slides were incubated for 5 minutes. Finally, after washing slides were covered with coverslip and immersed in Anti-Fade Fluoromount solution (Beyotime Institute of Biotechnology, Shanghai, China). PBMCs were observed by confocal microscope with laser scanner (PerkinElmer, Waltham, MA, USA) at 100 × magnification and digital pictures were taken using Nikon microscope software packages (Nikon, Tokyo, Japan).

### 2.9. Cell Proliferation Assay

Cell proliferation assay was performed in triplicate using cell counting kit-8 (CCK-8) assay reagent (Beyotime Biotechnology, Jiangsu, China) as reported previously [31]. Briefly, freshly rat PBMCs (1 × 10^6^ cells/mL) were seeded into 96-well plates and incubated with consecutive concentrations of rTs-SUCLA-β (10, 20 and 40 μg/mL), Concanavalin A (ConA), PBS and purified pET-32a protein (10 μg/mL) at 37 °C in a humidified atmosphere with 5% CO_2_ for 72 h. Before measuring the absorbance value (OD_450_) in micro plate reader (Thermo Scientific, Minneapolis, MN, USA), 10 μL of the CCK-8 reagent was added in each well for 4 h.

### 2.10. Cell Migration Assay

The migration assay was performed in triplicate using a Trans-well system (Merck-Millipore, Boston, MA, USA) that allows PBMCs to migrate through polycarbonate membrane (8 μm pour size) as described earlier [32]. Rat PBMCs (1 × 10^6^ cells/mL) were scattered into a 24-well plate (1 mL/well) and incubated with different concentration of rTs-SUCLA-β (10, 20 and 40 μg/mL), PBS and purified pET-32a protein (10 μg/mL) at 37 °C in a humidified atmosphere with 5% CO_2_ for 24 h.

### 2.11. Nitric Oxide Production Assay

Rat PBMCs (1 × 10^6^ cells/mL) were washed twice and 100 μL of cells were incubated with serial concentrations of rTs-SUCLA-β (10, 20 and 40 μg/mL), purified pET-32a protein and PBS in DMEM medium in 96-well plate at 37 °C in a humidified atmosphere with 5% CO_2_. Nitric oxide (NO) production assay was performed in triplicate by using Griess assay as the instructions given by Total Nitric Oxide Assay Kit (Beyotime Biotechnology, Jiangsu, China). The absorbance values of colored solution were measured using microplate reader at 450 nm (OD_540_) and converted these values to micro moles per liter (μmol/L) with standard curve obtained by adding 0–80 (μmol/L) of sodium nitrate in fresh culture media. This experiment was performed in three times individually.

### 2.12. Determination of Phagocytic Activity

The effect of rTs-SUCLA-β on the monocyte phagocytic ability in rat PBMCs was analyzed by Fluorescein Isothiocyanate (FITC)-dextran intake into the cells [33]. Freshly rat PBMCs (1 × 10^6^ cells/mL) were harvested into 24-well plates and incubated with consecutive concentrations of rTs-SUCLA-β (10, 20 and 40 μg/mL), PBS and purified pET-32a protein (10 μg/mL) at 37 °C in a humidified atmosphere with 5% CO_2_ for 48 h. After washing, cells were transferred into 1.5 mL tube in 100 μL PBS, and then added same volume of FITC-dextran (Sigma, St. Louis, MO, USA) in dark and incubated at 37 °C for 1 h. Each experiment was performed individually in triplicate.

### 2.13. Cell Apoptosis Assay

Freshly rat PBMCs (1 × 10^6^ cells/mL) were cultured into 24-well plates and incubated with consecutive concentrations of rTs-SUCLA-β (10, 20 and 40 μg/mL), PBS and purified pET-32a protein (10 μg/mL) at 37 °C in a humidified atmosphere with 5% CO_2_ for 24 h. After washing cells were re-suspended in binding buffer Annexin V-FITC (Miltenyi Biotec, Bergisch Gladbach, Nordrhein-Westfalen, Germany) was added to the cells and incubated for 15 min in dark at room temperature [34]. Flow cytometer (BD Biosciences, San Jose, CA, USA) was used after addition of propidium iodide (PI, Sigma-Aldrich, Shanghai, China) to the cell suspension.

### 2.14. Effect of rTs-SUCLA-β on Cytokines Expression In Vivo

To determine the effects of rTs-SUCLA-β protein on cytokines expression, SD rats (*n* = 30) were divided into three groups, group 1 (*n* = 10), group 2 (*n* = 10) and group 3 (*n* = 10). Pre-immune serum samples were collected one day prior to the first immunization by tail bleeding. Group 1 was intraperitoneal injected with 0.3 ng rTs-SUCLA-β in 0.5 mL PBS, group 2 with 0.3 mg purified pET-32a protein in 0.5 mL PBS and group 3 with 0.5 mL PBS. Sera samples were collected from all rats 1–6 days post immunization and the dynamics of gamma interferon-γ (IFN-γ), interleukin-9 (IL-9), transforming growth factor beta (TGF-β), interleukin-4 (IL-4) and interleukin-17 (IL-17) were evaluated by commercially available ELISA kit (Hengyuan Biological Technology, Shanghai, China).

### 2.15. Immune Protective Effects of rTs-SUCLA-β In Vivo

To investigate the immune protective effects of rTs-SUCLA-β, SD rats (*n* = 60) were divided into three groups, the experimental group (*n* = 20), vector group (*n* = 20) and control group (*n* = 20). Recombinant Ts-SUCLA-β protein (25 µg) was mixed equally with Freund’s complete adjuvant and subcutaneously injected to experimental group. After two weeks, one more protein dose mixed equally with Freund’s incomplete adjuvant was injected. Similarly, purified pET-32a protein (25 µg) was injected in vector group and control group was kept untreated. After 7 days of the last immunization all groups were infected with 200 *T. spiralis* ML by gavage. At 7 days post infection (DPI) rats (*n* = 10) from each group were euthanized to collect intestines. Longitudinally cut intestines were tiled on three layers of gauze in normal saline beaker, covered with an aluminum file and incubated at 37 °C for 4 h. Finally, adult worms were collected from the bottom of beaker and counted. Moreover, at 35 DPI remaining rats (*n* = 10) from each group were euthanized and MLs were collected by artificial digestion method as described above. Moreover blood samples were collected weekly from 0 days post immunization (DPIm) to 56 DPIm and to determine the immunoglobulin G levels (IgG, IgG1 and IgG2) enzyme-linked immunosorbent assay (ELISA) was performed (ELISA kits; Abcam, Cambridge, UK).

### 2.16. Statistical Analysis

The statistical analyses were performed by using the GraphPad Premier 6.0 software package (GraphPadPrism, San Diego, CA, USA). The differences between groups were compared by one-way ANOVA, followed by a Tukey test with (* *p* < 0.05, ** *p* < 0.01 and *** *p* < 0.001) [35]. Cell Quest Software was used to analyze the results of flow cytometry [36].

## 3. Results

### 3.1. Molecular Cloning and Sequence Analysis of Ts-SUCLA-β Gene

The fragment size of Ts-*SUCLA-β* gene (1353 bp) was detected between 2000 and 1000 (Figure S1A) and confirmed by digestion of restriction enzymes (*BamH*I and *Xho*I) using agarose gel electrophoresis and sequence analysis. The results indicated the successful insertion of Ts-*SUCLA-β* gene into frame of vector pET-32a (Figure S1B). Bioinformatics online tools predicted that no transmembrane region (Figure S2A) or signal peptide (Figure S2B) was found in Ts-SUCLA-β.

### 3.2. Expression, Purification and Immunoblotting of rTs-SUCLA-β

**Figure 1:**
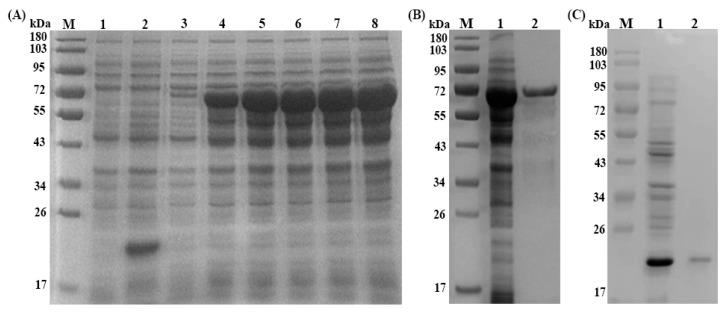
Expression and purification of rTs-SUCLA-β. Lane M: standard protein molecular weight marker. (**A**) Expression of rTs-SUCLA-β induced with IPTG. Lane 1: empty expression vector before induction; Lane 2: empty expression vector induced for 5 h; Lane 3: recombinant expression vector before induction; Lanes 4–8: recombinant expression vector induced for 1 to 5 h. (**B**) rTs-SUCLA-β was purified by Ni-NTA column. Lane 1: recombinant protein before purification; Lane 2: recombinant protein after purification. (**C**) Expression of purified his-tagged pET-32a. Lane 1: His-tagged protein before purification; Lane 2: His-tagged protein after purification.

**Figure 2:**
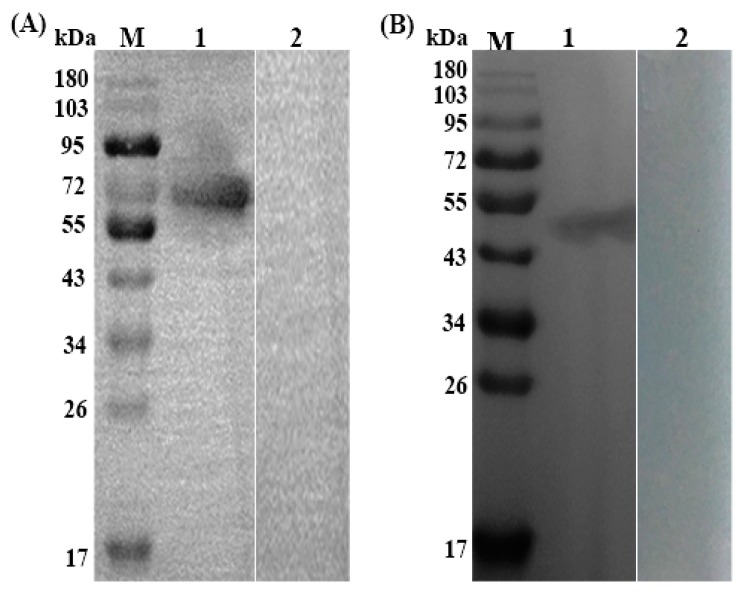
Western blot analysis. Lane M: standard protein molecular weight marker. (**A**) Western blot analysis of rTs-SUCLA -β. Lane 1: serum sample from *T. spiralis* experimentally infected mice; Lane 2: serum sample from normal mice; (**B**) Western blot analysis of ML protein. Lane 1: serum sample from SD rats containing polyclonal antibodies against rTs-SUCLA-β; Lane 2: serum sample from normal SD rats.

Recombinant plasmid of Ts-SUCLA-β (pET-32a + Ts-SUCLA-β) was induced with IPTG and analyzed by SDS-PAGE that showed highest concentration after five hours induction (Figure 1A). The expressed protein showed a molecular weight of about 65 kDa (Ts-SUCLA-β, 45.1 (fusion) pET-32a, 20) after purification (Figure 1B). Western blot analysis showed that rTs-SUCLA-β protein could be recognized by serum from mice experimentally infected with *T. spiralis* (Figure 2A, Lane 1) and be recognized by anti-rTs-SUCLA-β polyclonal antibodies (Figure 2B, Lane 1). However, no protein was detected with normal mice and rat sera (Figure 2A, B, Lane 2). The results indicated that rTs-SUCLA-β had good antigenicity and could be recognized by immune system of host.

### 3.3. Binding of rTs-SUCLA-β to Rat PBMCs

**Figure 3:**
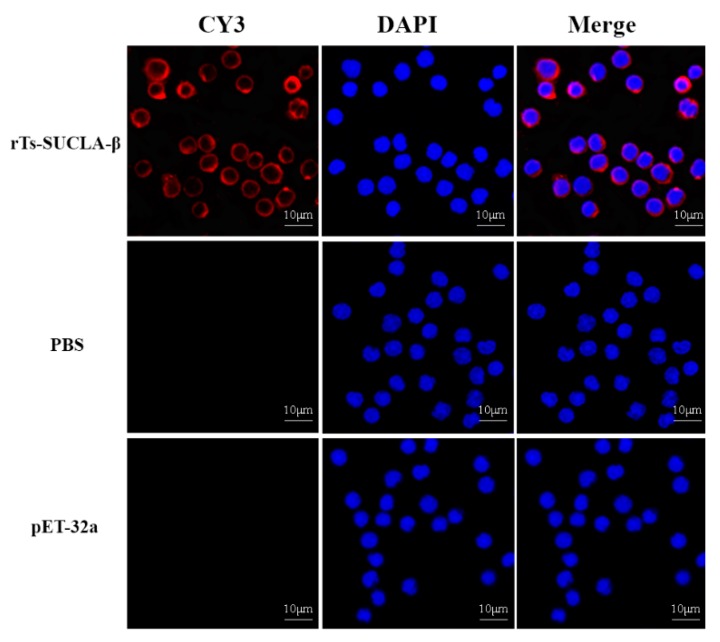
Binding of rTs-SUCLA-β with rat peripheral blood mononuclear cells (PBMCs) in vitro. After incubation of PBMCs with rTs-SUCLA-β, purified pET-32a protein or PBS, rat anti-rTs-SUCLA-β, anti-pET-32a protein or negative rat IgG (as first antibody) was added, followed by staining with Cy3-conjugated secondary antibody and DAPI. Red fluorescence on surface of cells showed target protein staining (Cy3) and nuclei of cells were visualized by DAPI (blue). No red fluorescence was observed in control groups.

The bindings of rTs-SUCLA-β to rat PBMCs were confirmed by immunofluorescence assay (IFA). DAPI was used to stain nuclei (blue fluorescence) and secondary antibody labeled with Cy3 to stain the cells (red fluorescence). Confocal microscopy results revealed that rTs-SUCLA-β was merged with the cell surface and showed red and blue combined fluorescence (Figure 3, upper section). Whereas no combined fluorescence was observed in cells treated with PBS and purified pET-32a protein (Figure 3, lower section).

### 3.4. Effect of rTs-SUCLA-β on Proliferation of PBMCs

**Figure 4:**
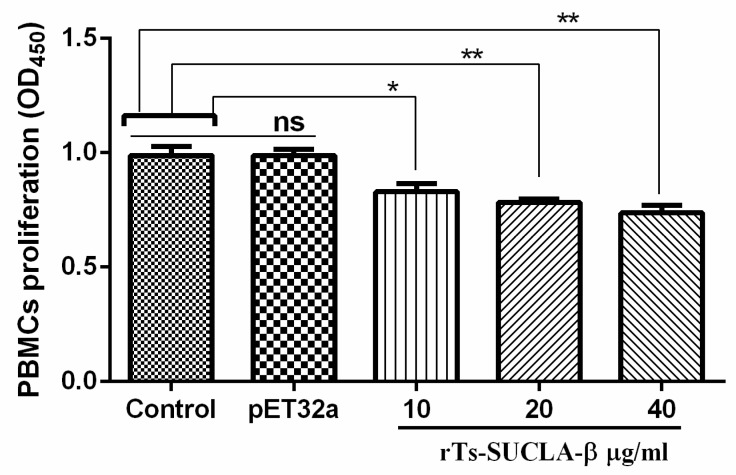
Effects of rTs-SUCLA-β on cell proliferation. PBMCs were treated with PBS (control), purified pET-32a protein and serial concentrations of rTs-SUCLA-β for 72 h. Cell proliferation index was calculated considering the Optical Density (OD_450_) values in PBS control as 100%. Significant differences was marked with * when *p* < 0.05, ** when *p* < 0.01 and ns (no significant difference). The data were analyzed from standard error mean of three independent experiments.

Cell counting kit 8 (CCK8) was used to evaluate the effects of rTs-SUCLA-β on the proliferation of PBMCs by performing cell proliferation assay. The results of this assay indicated that rTs-SUCLA-β produced significantly suppressive effect on rat PBMCs proliferation at 10 μg/mL (ANOVA, 20 μg/mL and 40 μg/mL (ANOVA, F _(5, 12)_ = 23.89, *p* = 0.0001), when compared to control (PBS). Whereas, no significant change was observed between purified pET-32a protein group and control group (ANOVA, F _(5, 12)_ = 23.89, *p* = 0.9999; Figure 4).

### 3.5. Effect of rTs-SUCLA-β on PBMCs Migration

**Figure 5:**
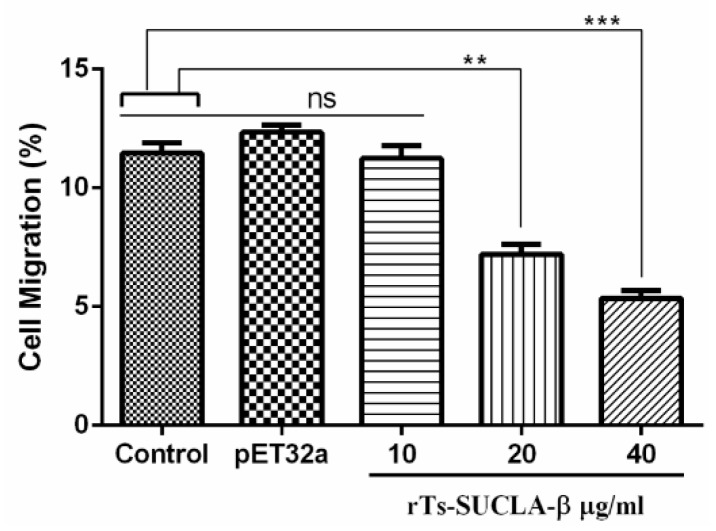
Effects of rTs-SUCLA-β on PBMCs migration. Cells were treated with PBS (control), purified pET-32a protein and serial concentrations of rTs-SUCLA-β. Then the random migration was determined. Statistically differences between standard error mean of three independent experiments (*n* = 5) were calculated using a one way ANOVA and marked with ** when *p* < 0.01 and *** when *p* < 0.001.

Cell migration assay was performed to evaluate the impacts of rTs-SUCLA-β on cell migration using Millicell® insert (Corning, NY, USA). The results indicated that percentage of migrated cells across the membrane in response to rTs-SUCLA-β was significantly deceased at 20 μg/mL (7.20 ± 1.220) and 40 μg/mL (5.36 ± 0.801) to that of control and purified pET-32a protein group (12.34 ± 0.921), but no effect was observed at 10 μg/mL (11.24 ± 1.48l Figure 5). 

### 3.6. Phagocytic Activity of rTs-SUCLA-β

**Figure 6:**
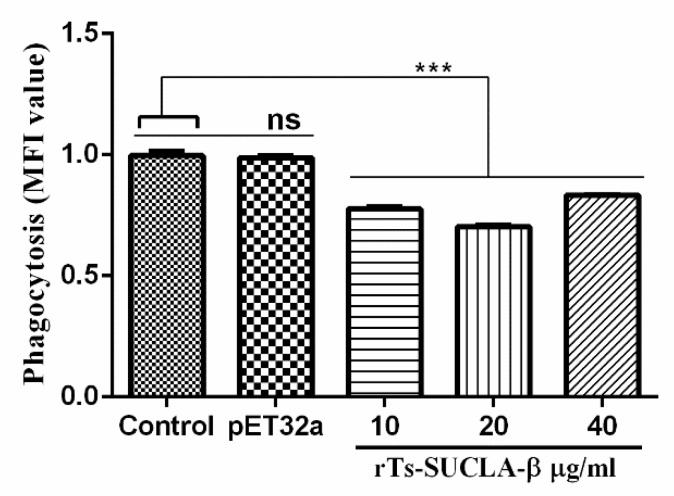
Effect of rTs-SUCLA-β on monocyte phagocytic activity in PBMCs treated with control (PBS), purified pET-32a protein and serial concentrations of rTs-SUCLA-β for 48 h, followed by incubating with Fluorescein Isothiocyanate (FITC)-dextran for 1 h. Phagocytic activity was tested based on FITC-dextran uptake by flow cytometry. Significant differences were calculated from the standard error mean of three independent experiments and marked with *** when *p* < 0.001.

Phagocytic abilities of monocytes in PBMCs treated with different concentrations of rTs-SUCLA-β were evaluated by cell phagocytosis assay using FITC-dextran. The results indicated that phagocytic activity was significantly decreased when rat PBMCs were treated with rTs-SUCLA-β at the concentration of 10 μg/mL, 20 μg/mL and 40 μg/mL (ANOVA, F _(4, 10)_ =162.1, *p* < 0.001). However, no significant difference was observed in purified pET-32a protein group (ANOVA, F _(4, 10)_ =162.1, *p* = 0.9593) when compared with control (PBS) group (Figure 6). 

### 3.7. Effect of rTs-SUCLA-β on PBMCs Viability and Apoptosis 

**Figure 7:**
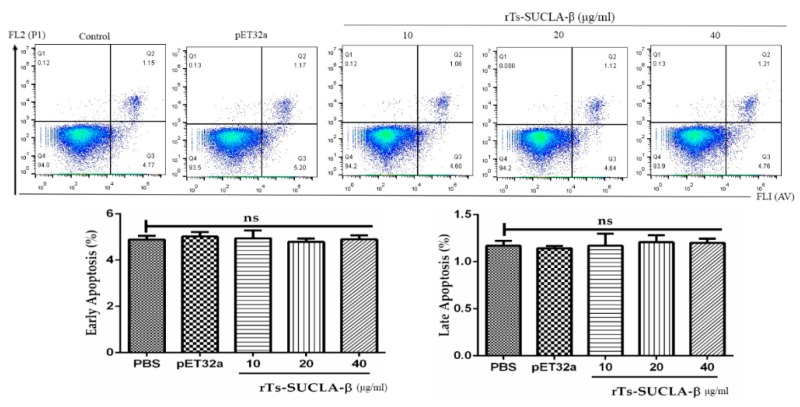
Effects of rTs-SUCLA-β on early and late apoptosis in PBMCs. Apoptosis of PBMCs was determined by flow cytometry after cells stained with Annexin V and propidium iodide. The percentages of cells with different staining patterns are shown in data statistics on the apoptosis of rat PBMCs (Q3 region of early apoptosis and Q2 region of advanced apoptosis). The data presented here are expressed as standard error mean of three independent experiments.

Cell viability assessed by means of the Trypan blue exclusion test was consistently >95%. Cell apoptosis assay was performed to assess the effects of rTs-SUCLA-β on PBMCs early and late apoptosis. The results showed no significantly difference between purified pET-32a protein and PBS (negative control) group. Furthermore, there was no significant effect (*p* > 0.05) of rTs-SUCLA-β on the early and late apoptosis of rat PBMCs at concentrations of 10 μg/mL, 20 μg/mL and 40 μg/mL was observed (Figure 7).

### 3.8. Effect of rTs-SUCLA-β on Cytokines Expression in Vivo

**Figure 8:**
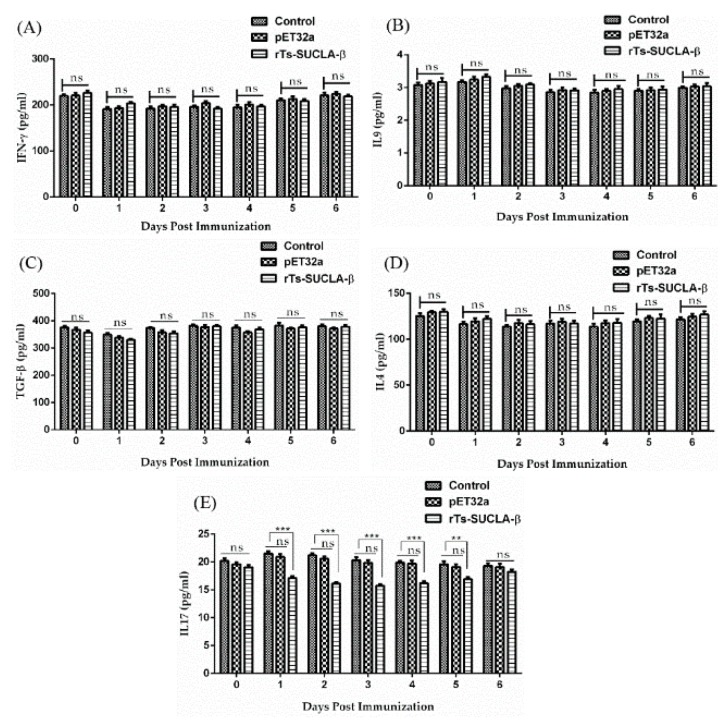
Effects of rTs-SUCLA-β on multiple cytokines expression in vivo. Rats were injected with rTs-SUCLA-β (PBS and purified pET-32a protein as controls). Serum from each rat was collected before injection (Day 0), 1, 2, 3, 4, 5 and 6 days after injection. Expression levels of IFN-γ (**A**), IL-9 (**B**), TGF-β (**C**), IL-4 (**D**) and IL-17 (**E**) were tested by ELISA. Significant differences were marked with ** when *p* < 0.01, *** when *p* < 0.001 and ns when non-significant. The data are expressed as the standard error mean with *n* = 10 in each group.

Serum samples collected at different days from SD rats of experimental (treated with rTs-SUCLA-β), positive control (treated with purified pET-32a protein) and negative control (PBS) groups were used to evaluate the effects of rTs-SUCLA-β on cytokines expression by ELISA. Results indicated that no significant effects on the secretion of IFN-γ cytokine was observed in the sera collected from rats of all groups at different days (Figure 8A). Similarly, IL-4, IL-9 and TGF-β cytokines did not show significant difference in the secretions at different days in all three groups treated with rTs-SUCLA-β, purified pET-32a protein and PBS (Figure 8B–D). While expression levels of IL-17 in PBS and purified pET-32a protein groups were not significantly different. On the other hand, decreased secretion effect of IL-17 was observed in rTs-SUCLA-β-treated experimental group at day 1, 2, 3, 4 and 5 (*p* < 0.001), while no significant effect (*p* > 0.05) on the secretion of IL-17 was observed in the rat sera at prior to injecting dose (day 0) and day 6 (Figure 8E). 

### 3.9. Parasite Burden Assessment

**Figure 9:**
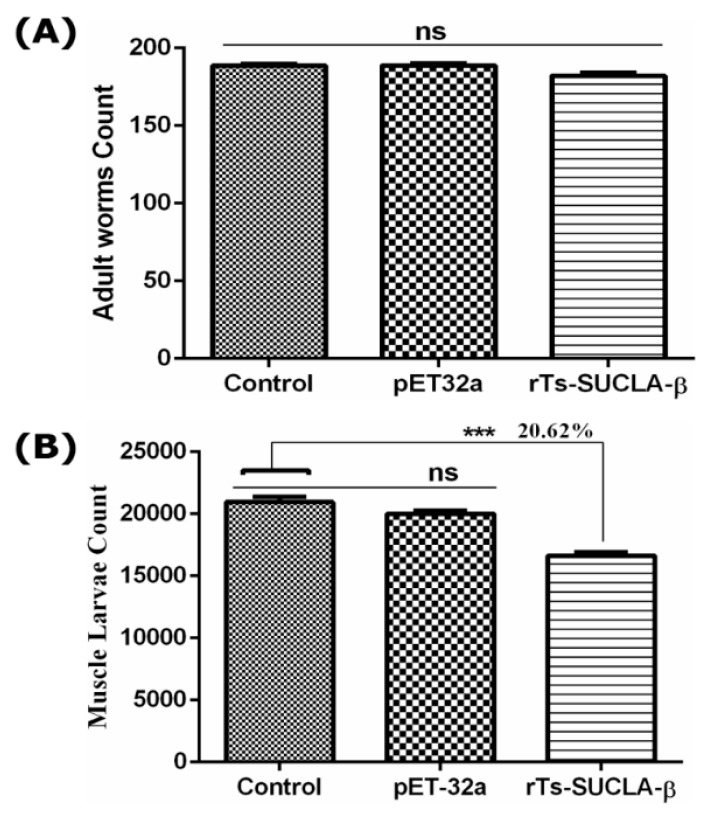
Parasite reduction in treated rats following challenge with 200 *T. spiralis* larvae per rat. (**A**) Adult larva count. No significant reduction of adults was observed between the groups. (**B**) Muscle larva burden reduction. The rTs-SUCLA-β showed 20.62% reduction of ML larva as compared to control. The purified pET-32a protein group and control group were non-significant. The results are presented as mean standard error of 10 mice per group. Significant differences was marked with *** when *p* < 0.001.

To evaluate the protective effects of rTs-SUCLA-β SD on adult worms, intestines were collected from rats at 7 DPI. Counting results showed no significant reduction of adult worms between experimental group, vector group and control group (Figure 9A). Moreover, ML were collected by artificial digestion at 35 DPI and counted. Significant reduction of infective muscle larva was observed in rTs-SUCLA-β treated group (F _(2, 27)_ = 43.65, *p* < 0.001) as compared to control group (Figure 9B). While no significantly difference was observed in vector group and control group (*p* > 0.05).

### 3.10. Antibody Detection 

**Figure 10:**
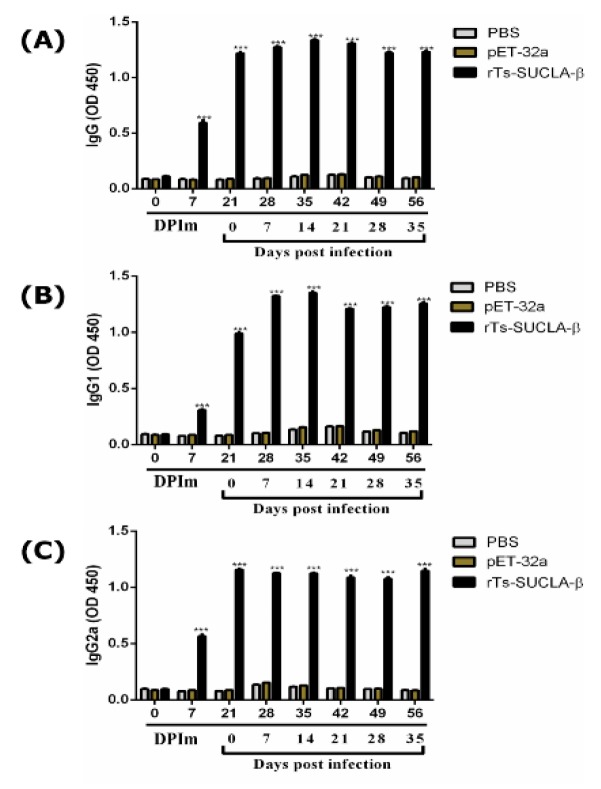
Detection of anti-rTs-SUCLA-β serum IgG by ELISA. (**A**) detection of IgG level. (**B**) Detection of IgG1 level. (**C**) Detection of IgG2a level. The values shown for each group are the mean standard error of the antibody levels (*n* = 10). The numbers 0–56 are days post immunization and 0–35 are days post infection of *T. spiralis*. Significant differences was marked with *** when *p* < 0.001.

Detection of immunoglobulin level (IgG, IgG1 and IgG2a) was performed by ELISA. Results demonstrated that the concentrations of IgG (Figure 10A), IgG1 (Figure 10B) and IgG2a (Figure 10C) antibodies were significantly increased after challenging the *T. spiralis* infection from 21 DPIm till 56 DPIm (0–35 DPI) in rTs-SUCLA-β group (*p* < 0.001) as compared to control. Moreover, at 0 DPIM no significant difference was observed between all groups. Furthermore, at 7 DPIm increased level of all three IgGs was observed in rTs-SUCLA-β group. While level of IgG, IgG1 and IgG2a were non-significant in purified pET-32a protein and control group from 0–56 DPIm (*p* > 0.05).

## 4. Discussion

Due to immunomodulatory properties of excretory and secretory products from infectious agents [37] especially helminths [38] may prevent autoimmune diseases. Multiple sclerosis infection with *T. spiralis* reduced the severity of experimental autoimmune encephalomyelitis development. In this study sequence analysis of Ts-SUCLA-β showed open reading frame of 450 amino acid (aa) and a conserved domain of succinyle-CoA synthetase superfamily, which is supposed to mainly coenzyme metabolism and transport the function domain. The aa sequence of Ts-SUCLA-β showed high homology with SUCLA-β of the *Trichinella* spp. In gene engineering, the most commonly expression system used for production of protein is the *E. coli* expression system. Gene SUCLA2 encoding a 65 kDa protein of *T. spiralis* was cloned successfully and expressed in *E. coli* using a pET-32a expression vector. Purified rTs-SUCLA-β was used to produce anti-rTs-SUCLA-β antibodies in mice. Furthermore, rTs-SUCLA-β was also recognized natively in ES antigens of ML found in sera of experimentally *T. spiralis* infected rats. Immunoblotting results revealed successful production of anti-rTs-SUCLA-β antibodies and indicated that rTs-SUCLA-β is one component of the ES protein from *T. spiralis* (Figure 2). Results of current study are in line with results of previous study [39]. 

The biological study of important parasites is based on the identification and development of novel drug targets [40]. In order to survive *T. spiralis* has ability to suppress the host immune response against itself, meanwhile it also suppresses the immune responses to allergens and auto-antigens, prevents malignant cell expansion [9] and modulates the development of autoimmune diseases [41]. Reports indicated that chronic helminth infection could be beneficial for both parasite and host. External immune-modulator from parasite might avoid or control the development of pathological immunity and excessive inflammatory responses for the host [42]. In acute inflammation, different types of cells including monocytes, lymphocytes, neutrophils and macrophages have been implicated [28]. Well-established infection of *T. spiralis* produces cysts in infected muscles from where larvae constantly release ESPs into the circulation and stimulate the host immune system [3]. ESPs are molecules secreted in vivo or in vitro by the worms and involve in complex functions of host parasite interactions. In the current study, the effects of one of the ML ESPs, Ts-SUCLA-β on some functions of rat PBMCs were firstly investigated. The results indicated that Ts-SUCLA-β presented suppressive potential on the PBMCs. Cell proliferation and migration are important processes that are related to immune response of the body and necessary for cells to participate effectively in host defense against pathogen invasion [34]. However, phagocytosis is critical for the clearance of the remnants of completed programmed cell death, opsonized pathogens and necrotic cell debris [43,44,45]. In this study, cell proliferation, migration and monocyte phagocytosis in rat PBMCs were significantly inhibited or decreased by rTs-SUCLA-β. These results suggested that rTs-SUCLA-β might inhibit the host immune responses. Nitric oxide production is a significant immune-mediator and plays important role in immunoregulation in various infections by mediating host protection or by suppressing the growth [46]. Previously, IL-17 involvement in T cell-mediated NO production was reported [47]. In the present study no immune-modulating effects of the rTs-SUCLA-β on the NO production by rat PBMCs was evaluated.

Cytokines secreted by monocytes and lymphocytes are deeply associated with immune (Th1 and Th2) and inflammatory responses, which perform vital immuno-regulatory functions [48]. In this study the effects of rTs-SUCLA-β on the production of different cytokines, IL-4, IL-9, IL-17, TGF-β IFN-γ and IL-17 in immune mice were evaluated. The results of this study showed no significant difference in the production of IFN-γ, IL-9, TGF-β and IL-4 cytokines at different days of the first week after challenge. Th1 immune response is characterized by the production of pro inflammatory cytokine such as interferon gamma [17], while Th2 is characterized by the production of interleukin-4 (IL-4) cytokine [49]. TGF-β is an important pleiotropic cytokine with potent immunoregulatory properties and is essential for the maintenance of immunological self-tolerance. Cytokines production, IL-4 and TGF-β promote the production of IL-9-secreting cells (T lymphocytes) [50]. IL-9 production is associated with the Th2 phenotype so this is the reason of unchanged production of IL-9. IL-17, known as the inflammatory factor in tissues, was reported to be an important cytokine against parasitic infections and multiple functional [51]. Th17 cells are concerned with the production of IL-17 and regulation of inflammation [52]. Moreover, in this study, we found that injection of rTs-SUCLA-β significantly reduced the secretion of IL-17 in immunized rats at day 1, 2, 3, 4 and 5 days post immunization while no significant effect on the secretion of IL-17 was observed in the rat sera at prior to injecting dose (day 0) and day 6. The rTs-SUCLA-β protein blocked the production of IFN-γ and IL-4. Production of IL-17 and IL-9 is inversely proportional to the production of IFN-γ and IL-4 cytokines [13]. IL-4 and TGF-β promote the production of IL-9 but both were also unaffected after the injection of rTs-SUCLA-β. Previous study indicated that TGF-β induces IL-9 production from Th17 Cells [53] but in our study TGF-β was static. This may be the possible reason for the suppression of IL-17. Previous study reported increased production of Th17 cells in the absence of TGF-β signaling in vivo [15]. In our study down regulated expression of IL-17 was observed when TGF-β expression was static. 

Moreover, involvement of IL-4 in PBMCs proliferation has been reported previously [2]. In this study production of IL-4 was recorded unchanged and this may be a possible reason of decreased PBMCs proliferation and associated functions. Both in vitro and in vivo results indicated that recombinant SUCLA-β from *T. spiralis* could down-regulate the immune functions of the host. Previously, some researches verified that infection of *T. spiralis* indicated protection of agouti rat against experimental autoimmune encephalomyelitis [3], ameliorating autoimmune diabetes in nonobese diabetic mice [54] and decreasing allergic airway reactivity and inflammation response [55]. One possible explanation is that the helminths and their produced immunomodulatory products directly modulate the host immune system to reduce anti-parasite immunity [56,57]. On the other hand, the long co-evolutionary history of helminths and their hosts has resulted in many parasites being relatively well tolerated and even contributing through their subtle dampening of inflammation to an optimal immunological balance [58]. Thus, to promote its own survival in the host during a chronic infection, a parasite may limit pathology by significantly affecting the host’s fitness to avoid serious collateral damage to its own tissues, a host may attenuate its immune responses to the parasite [59]. The rTs-SUCLA-β induced regulatory network it may be used as an immuno-modulator. Future studies will need to explore the effects of IL-17 inhibition by using rTs-SUCLA-β as immunosuppressive agents in clinically more relevant models. Furthermore, in this study protective effects of rTs-SUCLA-β were evaluated in rats. The rTs-SUCLA-β did not reduce the production of adult worms after infection of *T. spiralis* ML but after 35 DPI production of new generation ML were reduced. One of the possible reasons is that after production of newborn larva in intestine, circulatory system carried them to different organs but anti-*Trichinella* vaccine potentially prevented the intestinal *T. spiralis* to enter the striated muscles and develop into mature muscle larvae [9]. 

## 5. Conclusions

In conclusion, the *SUCLA-β* gene was successfully cloned and the recombinant Ts-SUCLA-β protein was expressed. Western blot assay demonstrated that rTs-SUCLA-β is one of the active proteins of ML that could be recognized by serum from mice experimentally infected with *T. spiralis* and by anti-*T. spiralis* generated antibodies. These findings showed that, the interaction of rTs-SUCLA-β with host cells decreased the production of IL-17, monocyte phagocytosis, migration and cell proliferation of PBMCs. However, no significant effect of rTs-SUCLA-β was observed on the secretion of IFN-γ, IL-9, TGF-β, IL-4, NO production and apoptosis of PBMCs. The role of IL-17 in vaccine-induced immunity against parasites is underexplored. Some studies suggest that IL-17 could be a potential target in the design of effective vaccines against parasites [60]. In contrast, IL-17 has been implicated in tissue pathology associated with parasitic disease [61]. In this study rTs-SUCLA-β (25 µg) showed 20% reduction of ML with strong effects on the IgGs production. Reduction rate may be improved by increasing the injected dose of rTs-SUCLA-β.

Our findings will provide valuable bases to better understand the biology of *T. spiralis* and host interaction and may contribute to reduce the chronic inflammation associated with autoimmune diseases and graft rejection by suppressing inflammatory IL-17 immune response. Therefore, further research targeting IL-17 in vaccine strategies should be carefully studied. Moreover, rTs-SUCLA-β reduced 20% of the new generation of *T. spiralis* ML in vivo so it may be a potential molecular target by improving the reduction rate for controlling *T. spiralis* infection.

## 6. Ethics Approval and Consent to Participate

The study was conducted following the guidelines of the Animal Ethics Committee, Nanjing Agricultural University (NAU), China. All experimental protocols were approved by the Science and Technology Agency of Jiangsu Province (Approval ID: SYXK (SU) 2010-0005).

## Supplementary Materials

The following are available online at https://www.mdpi.com/2076-393X/7/4/167/s1, Figure S1: Cloning of SUCLA-β gene and identification of the recombinant plasmid (pET-32a (+) Ts-SUCLA-β); Figure S2: Membrane protein prediction and N-terminal signal peptide prediction of Ts-SUCLA-β.

## Abbreviations

SUCLA-β	Succinate Coenzyme A ligase beta-like protein
PBMCs	Peripheral blood mononuclear cells
Th	T helper
IL	Interleukin
TGF-β	Transforming growth factor-beta
ML	Muscle larvae
ESPs	Excretory and secretory products
NBL	New born larvae
ORF	Open reading frame
CDS	Conserved domain sequences
LB	Luria Bertini
PVDF	Polyvinyl Difluoride
IPTG	Isopropyl-b-D-thiogalactopyranoside
SDS-PAGE	Sodium dodecyl sulfate-polyacrylamide gel electrophore
RPMI	Roswell Park Memorial Institute
DPI	Days post infection
DPIm	Days post immunization
ELISA	Enzyme-linked immunosorbent assay
