# Supplementary material for: Succinate Coenzyme A Ligase Beta-Like Protein from Trichinella spiralis Suppresses the Immune Functions of Rat PBMCs In Vitro and Inhibits the Secretions of Interleukin-17 In Vivo
Source: Vaccines (Basel). 2019 Nov 2;7(4):167. doi: 10.3390/vaccines7040167 (PMC6963543; doi:10.3390/vaccines7040167)
Supplement: Supplementary file 1 [file vaccines-07-00167-s001.pdf]

Supplementary Materials:

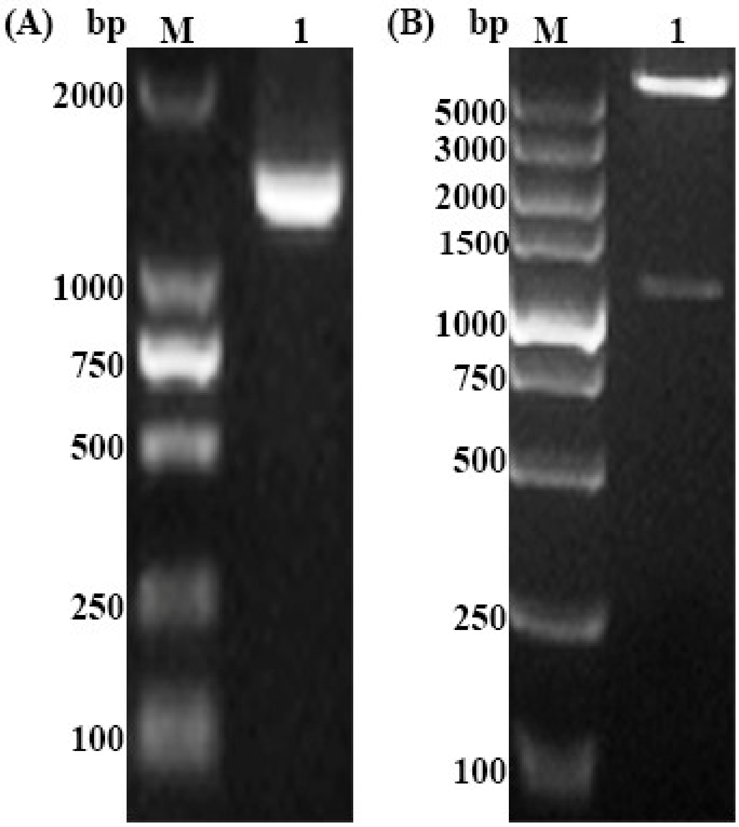

Figure S1. Cloning of SUCLA-β gene and identification of the recombinant plasmid (pET-32a (+) Ts-SUCLA-β). Lane M: DNA marker; (**A**) Lane 1: Amplified SUCLA2 gene by PCR. (**B**) Lane 1: Recombinant plasmid (pET-32a (+) Ts-SUCLA-β) were digested with *BamH* I and *Xho* I.

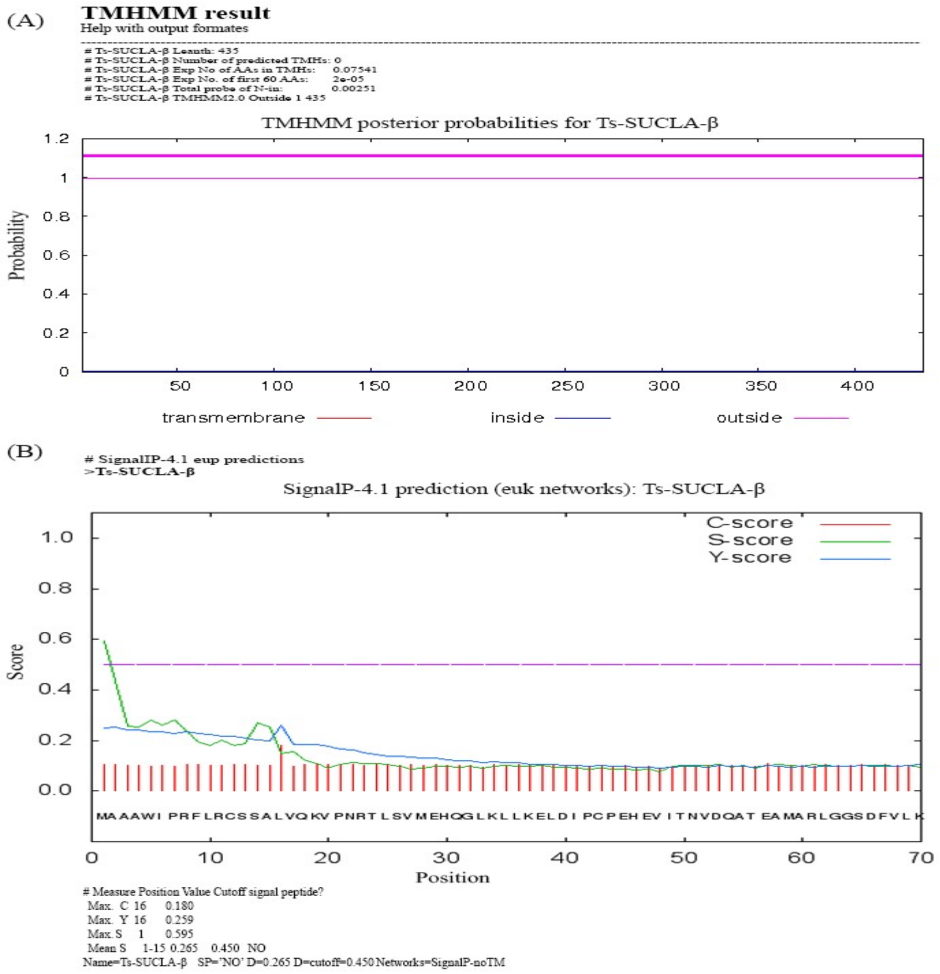

Figure S2. Membrane protein prediction and N-terminal signal peptide prediction of Ts-SUCLA-β. (**A**) The result predicted by online tool TMHMM showed that Ts-SUCLA-β have no transmembrane region. (**B**) Online tool SignalP predicted that no signal peptide was found in Ts-SUCLA-β.
